# Perceptual information supports transfer of learning in coordinated rhythmic movement

**DOI:** 10.1007/s00426-020-01308-1

**Published:** 2020-03-04

**Authors:** Daniel Leach, Zoe Kolokotroni, Andrew D. Wilson

**Affiliations:** Department of Psychology, Leeds School of Social Sciences, Leeds Beckett University, Leeds, UK

## Abstract

In this paper, we trained people to produce 90° mean relative phase using task-appropriate feedback and investigated whether and how that learning transfers to other coordinations. Past work has failed to find transfer of learning to other relative phases, only to symmetry partners (identical coordinations with reversed lead–lag relationships) and to other effector combinations. However, that research has all trained people using transformed visual feedback (visual metronomes, Lissajous feedback) which removes the relative motion information typically used to produce various coordinations (relative direction, relative position; Wilson and Bingham, in Percept Psychophys 70(3):465–476, 2008). Coordination feedback (Wilson et al., in J Exp Psychol Hum Percept Perform 36(6):1508, 2010) preserves that information and we have recently shown that relative position supports transfer of learning between unimanual and bimanual performance of 90° (Snapp-Childs et al., in Exp Brain Res 233(7), 2225–2238, 2015). Here, we ask whether that information can support the production of other relative phases. We found large, asymmetric transfer of learning bimanual 90° to bimanual 60° and 120°, supported by perceptual learning of relative position information at 90°. For learning to transfer, the two tasks must overlap in some critical way; this is additional evidence that this overlap must be informational. We discuss the results in the context of an ecological, task dynamical approach to understanding the nature of perception–action tasks.

## Introduction

This experiment is about how people learn to produce a novel bimanual coordinated rhythmic movement (specifically, 90° mean relative phase) and whether this learning transfers to other mean relative phases. Previous work has found no such transfer, but all this work uses transformed visual feedback during training which removes the relative motion information variables known to support the perception of relative phase (relative direction, relative position; Wilson & Bingham, 2008; Wilson, Collins, & Bingham, 2005b). We trained ten people to produce 90° using more task-appropriate *coordination displays* and *coordination feedback* (Wilson, Snapp-Childs, Coats & Bingham, 2010b), in which these variables remain available. We replicate the finding that learning 90° entails learning to perceive relative phase with a new information variable (from relative direction to relative position; Wilson & Bingham, 2008), and then, for the first time, show that this perceptual learning supports substantial transfer of learning 90° to both 60° and 120°.

We will first briefly review the current evidence about transfer of learning, which leads us to identify that the literature has yet to identify a suitable definition of task to account for the various results. We then introduce the ecological, task dynamical approach to defining perception–action tasks, explain it with reference to work on coordinated rhythmic movement, and use it to motivate the current study. We end by discussing how the results support this notion of task, which then allows us to start predicting and explaining learning and transfer results in the literature.

## Transfer of learning

Learning is a relatively permanent change in the behavioral repertoire of an organism caused by purposeful practice. When we study learning, we are interested in uncovering what changes in the organism to support this new behavior. One way to tackle this question is by looking to see how narrow or broad the effects of training are; do we just learn what we practiced, or does that learning transfer to other untrained tasks in any way? The way in which learning transfers, or fails to do so, allows us to map out tasks and which parts they share, or do not.

It does not seem to be the case that we have to learn every new task from scratch, so there is a basic expectation in the motor control literature that learning should transfer to at least some extent. Specifically, motor control theories predict that transfer will occur to the extent that ‘identical elements’ are present in the transfer and criterion tasks (Thorndike & Woodworth, 1901) and we should see transfer from training on parts of a task to the whole task if we have successfully decomposed the task into its ‘natural parts’ (Teague, Gittelman, & Park, 1994). Experimentally, we say there is transfer of learning when learning on one task (the *transfer task)* results in either similar patterns of change in performance on an untrained task (the *criterion task*), or *savings* in the time taken to learn the criterion task. Transfer can then vary in either direction, or magnitude, or both. Performance on the criterion task could increase (*positive transfer*) or decrease (*negative transfer*) as a function of practice on the transfer task, and the amount of performance change would be some percentage of the change in the transfer task (*percentage transfer*; see Schmidt & Young, 1986, for a detailed review).

Tasks can often involve similar capacities of the system (e.g. requiring ‘balance’ or ‘coordination’) and so we might expect transfer to be fairly common. However, the data actually suggest that transfer is surprisingly limited and, when it occurs, typically very small in magnitude (Schmidt and Young, 1986). Early research used multiple pursuit-rotor experiments in which the transfer task varied only by speed (RPM) from the criterion task, but these only showed a surprisingly low average transfer of 37% (Lordahl & Archer, 1958; Namikas & Archer, 1960). This transfer can be increased by increasing task difficulty (e.g. Siegel & Davis, 1980; Wang, Zhou, & Liu, 2013), but often transfer is better predicted by increasing similarity between the transfer and criterion tasks (e.g. Jeter, Dosher, Petrov, & Lu, 2009; Leonard, Karnes, Oxendine, & Hesson, 1970). For example, transfer can be quite high if the criterion task is the same action, but performed either with the opposing limb (bilateral transfer, e.g. Munn, 1932) or another effector (cross-effector transfer, e.g. Kelso & Zanone, 2002) than was trained. In general, however, the proportion of transfer remains small even if the tasks seem to require the same general capacities, such as balance.

The part-whole transfer literature is similarly mixed. Part-whole transfer is an attempt to decompose a task into its natural parts (subtasks), training in a subtask, and measuring the transfer to the whole task. Successful transfer is then evidence that the subtask is actually a natural part of the larger task. In general, findings are inconsistent among tasks (Schmidt & Young, 1986; Teague, Gittelman, & Park, 1994) and certain task types (discrete, continuous) are incredibly difficult to meaningfully decompose. The intuitive general capacities researchers decompose the task into (e.g. gross body equilibrium, dynamic flexibility, stamina, rate control, control precision, multilimb coordination) are not reliably shown to be natural parts of performance. For example, Serrien et al. (2017) observed minimal transfer between balancing on a slackline and balancing on a stable surface; ‘balancing’ is not a general purpose capacity on call for these different tasks.

The rather surprising fact, then, is that learning a perception–action task seems to be highly specific to that task, and transfer is only observed when the task demands are highly similar. It also seems clear that current models of what a task is have not yet successfully identified what the natural parts of a task are. In the next section, we will lay out an ecological approach to this question, and then apply it to understanding learning and transfer in the coordinated rhythmic movement.

## Ecological task dynamics

As we have just noted, current approaches to decomposing tasks into components are not working; the perception–action system is not composed of collections of generalized capacities such as ‘balance’ or ‘coordination’. There are two options at this point; either tasks cannot be meaningfully decomposed into parts and transfer of learning really is extremely limited, or they can be decomposed, if guided by a different heuristic which would lead to different components. We propose it is the latter. We will take an ecological, task-dynamics approach to understanding what a task is and what performance in that task is made of (Golonka & Wilson, 2012, 2019a; Wilson & Golonka, 2013). We will then apply that framework to the problem of when learning might transfer.

### The dynamical ecological environment

Task environments are best characterized in terms of dynamics, because this allows us to fully characterize both the motions (kinematics) and the forces causing those motions (kinetics) as tasks/events unfold in the world. When modeling a task, each candidate component must be described using dynamical variables. The state of those variables is then set with parameters, and the organisation of the system is captured by the form of the equation connecting the parameterised variables together. The behavior of the system then emerges as this particular task dynamic (the specific composition and organisation of the dynamical variables in the task) unfolds over time, and the parameters then implement one particular instance of the underlying dynamic.

This also means that tasks and events can only be uniquely identified at the level of dynamics (Bingham, 1995; Wilson & Bingham, 2001). For example, fly balls in baseball vary greatly in their specific motions (kinematics) but are all instances of the same underlying dynamic (projectile motion), just with varying parameters (release angle, height and velocity). They are only “the same” when considered dynamically. This provides us with a formal way to characterize when two tasks are the same or different, and it suggests that the challenge facing a learner is developing the ability to interact with a task at the level of dynamics. To do so, people must be able to perceive these dynamics (Bingham, 1988).

### Kinematic specification of dynamics

Perceptual information variables are higher order patterns in low-energy media such as light or the atmosphere. When energy (for example, light) interacts with the components of a dynamical task, the resulting structure in the light (the optic array) is a kinematic projection of that event into the array; the structure can be fully characterized in terms of its motions, with no reference to any forces. The kinematic information can therefore not be identical to the dynamical event, but it can still specify (map 1:1 to) that event (the *kinematic specification of dynamics*; Runeson & Frykholm, 1983) and, over time, we learn to use these kinematic patterns as information for the underlying dynamics (e.g. Wickelgren & Bingham, 2001). This ‘perceptual bottleneck’ (Bingham, 1988) means we do not have unmediated access to the task dynamics we need to perceive, and so to understand how we are interacting with those dynamics, we have to understand the form of the specifying information.

Applying task dynamics and the need for perception to the domain of learning, therefore, leads to the following research agenda to explain a pattern of observed behaviors (Wilson & Golonka, 2013). First, you must characterize your task with the appropriate dynamics. You must then use this characterization to identify the kinematic, ecological information created as the components implementing that dynamic interact with an energy media. Third, you should formalise this analysis in a dynamical model. Finally, you must test to see whether organism behavior can be predicted and explained by this analysis. This is the modern, dynamical formulation of the ecological approach to perception and action (Gibson, 1966, 1979; Turvey et al., 1981; Wilson & Golonka 2013), which we can now extend to address the question of transfer.

### Transfer of task dynamical learning

Learning is generally predicted to transfer to the extent that the two tasks overlap in some meaningful, structural way. Ecological task dynamics predicts that learning in one task will transfer to another task only if they are perceived to entail the same dynamics. Specifically, *learning to coordinate and control your behavior with respect to an information variable in the context of one dynamical task will support transfer of that learning if and only if the dynamics are the same and, therefore, the information those dynamics create is* (*a*) *the same and* (*b*) *still supports a functional interaction with those dynamics.*

This ecological task-dynamics analysis has so far been developed most completely in the context of coordinated rhythmic movement (Bingham, 2001, 2004a, 2004b, Golonka & Wilson, 2012, 2019b; Snapp-Childs, Wilson, & Bingham, 2011; Snapp-Childs, Wilson, & Bingham, 2015). The next section will briefly review that work and then set up the current study as the next step in the research programme.

## The task dynamics of coordinated rhythmic movement

A coordinated rhythmic movement is one in which a person is tasked with oscillating a limb at some mean relative phase to either another limb or a simulated oscillator. Phase is the angular measure of position within a cycle, and relative phase is simply the difference in phase between two oscillators. It is the appropriate dynamical variable to capture the coordination of the limbs; the question is, how is that dynamical variable perceived?

The primary clue comes from how coordination is structured with respect to relative phase. Humans show a specific pattern of coordination stabilities, first described experimentally by Cohen (1971) and Yamanishi, Kawato, & Suzuki (1979, 1980) and then studied in detail and modeled by Kelso (Haken, Kelso, & Bunz, 1985; Kelso, 1981, 1984, 1995). People can coordinate rhythmic movements either in-phase or anti-phase, with the former being more stable. Other coordinations are difficult or impossible to maintain, and small perturbations will make the person swiftly transition to one of the two stable coordinations. This pattern was originally defined and explained in terms of muscle homology (an egocentric frame of reference); moving in-phase entails using the equivalent muscles in each limb at the same time. However, the HKB pattern persists when coordination occurs in an external, allocentric frame of reference. Specifically, it is seen when the coordination is between people (Schmidt, Carello, & Turvey, 1990), between people and a display (Wilson et al., 2005a, 2005b) and even in perceptual judgments with no movement requirement (e.g. in vision: Bingham, Zaal, Shull, & Collins, 2001; Zaal, Bingham, & Schmidt, 2000; and in proprioception: Wilson, Bingham, & Craig, 2003). This suggests that the stability pattern emerges from the way we perceive coordination dynamics.

Bingham (2001, 2004a, 2004b; Snapp-Childs et al., 2011) applied an ecological task dynamical analysis to coordination (see Golonka & Wilson, 2012, 2019b for an extended review of this analysis process). The goal was to correctly characterize the dynamical structure of the task of coordinated rhythmic movement, and then to identify the resulting perceptual information about relative phase that people might use.

Dynamically, rhythmically moving human limbs are best characterized as damped mass springs (Feldman, 1986, 2011; Kay, Kelso, Saltzman, & Schöner, 1987; Kay, Saltzman, & Kelso, 1991), specifically phase-driven damped mass springs (Bingham, 1995, 2004a, 2004b). These must now be perceived. Each limb’s motion creates local optic flow traveling in the direction it is heading, e.g. from left to right or vice versa. Bingham (2001, 2004a, 2004b) noted that in the allocentric, visual frame of reference, the stability of the *relative* direction of these motions matched the HKB pattern. At 0°, the relative direction is consistent, and always that they are moving in the same direction at the same time; at 180°, the relative motion is still consistent but now always moving in the opposite direction at the same time; and at 90°, the relative direction is maximally variable (half the time, it is the same, half the time, it is opposite). Every other relative phase is uniquely specified by a particular relative direction pattern, and these optical patterns are available both when producing and observing coordinated rhythmic movements. Bingham, therefore, proposed that the relative direction of the optic flow is the specifying information variable that people use to perceive relative phase, and that this causes the observed pattern of movement stability. Relative speed was predicted to act as a noise term on the detection of this variable.

All the key predictions of the model have received empirical support. Movement stability is a function of perceptual stability (Wilson et al., 2005b) and perceptual training improves movement stability without movement practice (Bogaerts, Buekers, Zaal, & Swinnen, 2003; Wilson, Snapp-Childs, & Bingham, 2010). One paper failed to support the hypothesis that relative speed acts as a noise term (de Rugy, Oullier, & Temprado, 2008) but Snapp-Childs et al. (2011) corrected problems in the methodology and analysis in that paper and confirmed the model prediction.^1^ Finally, relative direction has been identified as the information most untrained participants use to perceive relative phase (Wilson & Bingham, 2008; Wilson et al., 2005a). Coordination stability looks the way it does because of the stability of the information we use to perceive and control that coordination.

Wilson & Bingham (2008) also tested the participants from Wilson et al. (2010) who had received extensive perceptual training at 90° and showed that this learning entailed switching to perceiving relative phase with a new variable, relative position. 90° is specified by this variable when one oscillator is at its maximum amplitude at the same time as the other is exactly halfway through its motion from side-to-side. This was identified by selectively perturbing relative position, altering the trajectory so that neither the endpoints nor the midpoints of the movement from side to side was consistent (see Wilson & Bingham, 2008 for more details, including plots of the resulting motions). Relative position and relative phase remained defined, but now the former no longer specified the latter. This disrupted trained performance at 90° in all participants, showing they were using relative position as information for relative phase and could not do the task when that no longer worked. Interestingly, this perturbation affected three untrained participants at 180° as well, suggesting that relative position can serve as information for relative phase beyond 90°. We will discuss this more below.

The basic insight into the model is that coordinated rhythmic movement is a perception–action task and that the ecological task dynamical analysis that decomposes the task into both dynamics and the associated information has been very successful in explaining the characteristic phenomena. The model does not currently include a specification of relative position and, therefore, does not yet explicitly explain the learning process. Part of the purpose of this paper is to improve our understanding of learning in this task and what relative position is to allow us to extend the model in the future.

### Transfer of learning in coordinated rhythmic movement

As noted above, the ecological task dynamical analysis predicts that learning in one context will transfer to another context to the extent that the information learned in the first supports successful behavior in the second. The value of coordinated rhythmic movement as an experimental task is that the task dynamics and associated information are tractable problems, and the resulting model can help us understand the existing patterns of learning and transfer of learning in coordination tasks.

The first round of coordination transfer studies only found evidence for bilateral and cross-effector transfer of learning. Learning a novel coordinated rhythmic movement (i.e. 90°) transferred only to the symmetry partner (270°; Zanone & Kelso, 1997). Given that symmetry partners are identical coordinations except for which limbs are leading and lagging, this is not transfer to a novel coordination; rather, it is another demonstration of bilateral transfer, which as previously mentioned is almost always positive and large in magnitude. Additionally, learning these relative phases in one set of limbs transfers to a different set of limbs (cross-effector transfer; Kelso & Zanone, 2002). This pattern accords well with the general findings that learning tends to be very specific to the trained outcome but less specific to the means of achieving that outcome (Schmidt & Young, 1986).

These transfer studies (and the many other learning studies in the field) have a limitation, however. Novel coordinations are hard to produce, because they are hard to perceive (e.g. Zaal et al., 2000). To make the to-be-learned relative phase perceptually clear, research has relied on transformed visual feedback, in which the continuous relative motion of the actions is turned either into discrete timing information (visual metronomes, Kelso & Zanone, 2002; Zanone & Kelso, 1997) or combined into a single continuous motion (Lissajous figures; Lee, Swinnen, & Verschueren, 1995). While effective for learning, these transformations change the underlying task dynamic. While the limb dynamics are similar, relative direction and relative position are not defined in the visual feedback and, therefore, cannot be used as information for relative phase, nor are they available to be learned. This has consequences. For example, people can swiftly use Lissajous feedback to stably produce any mean relative phase, i.e. they no longer show the HKB pattern (Kennedy, Wang, Panzer, & Shea, 2016; Kovacs, Buchanan, & Shea, 2009). While altering, the task dynamic can be experimentally useful if done on purpose, in this case, it means we can no longer apply the ecological task dynamical analysis implemented in the Bingham model to understand patterns of learning and transfer.

To solve this problem, Wilson, Snapp-Childs, Coats, et al. (2010) developed *coordination feedback*. This presents a display of two dots being moved either by a computer or a person. Whenever the coordination between the two dots is at the target relative phase +/− a customisable error range, the dots change colour from white to green. The display is ‘full cue’ (all the relative motion information variables remain defined and available) and so does not bias performance or learning by prescribing the options. The colour change is a task-dynamic-irrelevant variable (colour plays no role in coordination stability; Mechsner & Knoblich, 2004) and so it serves solely as a hot/cold cue about whether the current performance is correct or not. The error range can be set to suit the experiment; in our learning studies, we typically start at a large range (e.g. ± 30°) and then systematically reduce the range over sessions to drive continued improvement (typically down to ± 10°). Wilson et al. (2010) showed that this method produced learning at 90°, while a control group that saw the white dots but not the colour change feedback did not learn.

Our first application of this technique to the question of transfer of learning was Snapp-Childs et al. (2015) who used coordination feedback and trained participants to move at 90° either unimanually or bimanually. In the former, participants learned to move one dot at 90° to a computer controlled dot, while in the latter, they were in control of both dots. Crucially, the informational coupling remains the same. Participants learned their task, and this learning transferred to the other task. The percentage transfer was quite high (43–45%) and equal once the different stabilities of the two tasks was accounted for. Participants also all improved in a test of their visual discrimination of 90°. Snapp-Childs et al. (2015) concluded that the perceptual learning was identical in both tasks, that the learning supported successful coordination in both tasks, and that, therefore, transfer was possible and occurred.

## The current experiment

Snapp-Childs et al. (2015) showed that the ecological task dynamical analysis could guide the interpretation of transfer effects with reference to the underlying mechanism. In particular, transfer followed the information, despite the fact that unimanual and bimanual coordination tasks entail quite different limb dynamics, there is task dynamical structural overlap in the information used to control the coordination and transfer was observed. So far, however, there is no evidence of transfer of learning between relative phases other than symmetry partners, because all the existing work has used transformed visual feedback which removes the potential for informational overlap beyond symmetry partners.

We know from previous work that learning to perceive or move at 90° with coordination feedback entails switching to using relative position as information for relative phase. We also know that relative position is a viable information variable for relative phases other than 90° (e.g. 180°; Wilson & Bingham, 2008). The current study, therefore, extensively trained participants (*n* = 10) in bimanual 90° movements under full cue, task appropriate, coordination feedback conditions. We confirmed that this learning entailed switching to relative position, and then asked whether that learned variable can support stable coordination at other relative phases. We assessed their coordination stability in three assessment sessions (Baseline, Post-Training and Retention) at seven relative phases; 0°, 180° and 90° but also 30°, 60°, 120° and 150°, looking for learning at 90° and then any transfer of that learning. We also assessed their visual discrimination of 90° in the three assessment sessions using a two-alternative forced choice staircase procedure to demonstrate again that their change in movement stability was being enabled by a change in perceptual ability. Finally, we assessed their Post-Training and Retention visual discrimination of 90° using the relative position perturbation procedure (Wilson & Bingham, 2008) to confirm that this was the information variable participants had switched to improve their perception and production of 90°.

We predicted that the judgment data would mirror the learning data in the action task (as in Snapp-Childs et al., 2015). Specifically, learning to move at 90° would lead to improved visual discrimination of 90°. We predicted that after training, perturbing relative position would disrupt trained 90° judgment performance (as per Wilson and Bingham, 2008). As this is the first time that transfer has been investigated using coordination feedback, we have no explicit predictions regarding the magnitude or location of the transfer; however, given that relative position can be used as information at non-90° relative phases, we expected to see at least some transfer.

## Methods

This experiment’s design and analysis plan was preregistered (Leach, Wilson & Kolokotroni, 2016; http://osf.io/u72j9). We originally intended to distinguish between learners and non-learners in our analysis based on whether or not participants made it all the way through to the hardest training session parameters. 4 of the ten included participants were classified by this criterion as ‘non-learners’; however, we found no evidence of any differences in their actual performance^2^ which suggests our progression criterion was not appropriate, and so we, therefore, ran the main analysis on the entire cohort.

### Participants

Fourteen adults participated in this study, four of whom chose not to complete the entire procedure leaving a total of ten participants (female = 7; 18–29 years old, M = 21.3). Participants were recruited from the local area of Leeds, UK.

All participants were free from known neurological defects or motor disabilities, had normal or corrected-to-normal vision and were right handed (measured with the Edinburgh Handedness Inventory; Dragovic, 2004; Oldfield, 1971). All participants were naïve to the experimental questions and declared no previous engagement with this type of task. Prior to training, all participant’s relative phase production matched the predefined criterion for participation (see “Criteria”). All participants were paid £15 upon competition of the study. Ethical approval was granted by the Psychology Ethics Committee at Leeds Beckett University, UK.

### Design

All participants performed two types of experimental task; coordinated rhythmic movements (*Action*) and two-alternative forced choice (*Judgments*).

For the Action tasks, there were two within-subject variables. The first is Session (three levels; Baseline, Post-Training, Retention). These sessions were referred to as assessment sessions, to distinguish them from the training sessions. The second was Target Phase (seven levels; 0°, 30°, 60°, 90°, 120°, 150° and 180°). The dependent variable was the Proportion of Time on Target phase ± 20° (PTT20), a valid measure of performance (see Snapp-Childs et al., 2011, 2015 for explicit comparisons of this to other commonly used measures, which motivates us to prefer PTT20).

For Judgment tasks, there was one within-subjects variable, Session (3 levels; Baseline, Post-Training, Retention). The dependent variable was the estimated threshold to identify 90° in the Judgment tasks (the lower the threshold, the greater the ability to discriminate 90°).

## Materials

All sessions were performed on a Windows PC with a 24″ Dell monitor located approximately 70 cm from the participants. The computer presented a display of two white dots (~ 15 mm), separated vertically (~ 35 mm), that moved horizontally across a black background (screen refresh rate 60 Hz, resolution 1920 × 1080). The motion of both dots was centred at the screen centre with an amplitude of 300 pixels (~ 115 mm). All displays were presented, controlled and recorded by a custom MATLAB toolbox written by ADW incorporating the Pyschtoolbox (Brainard, 1997; Kleiner et al., 2007; Pelli, 1997, http://psychtoolbox.org). Matlab 2014b was used to record and analyze the data.

**Fig. 1:**
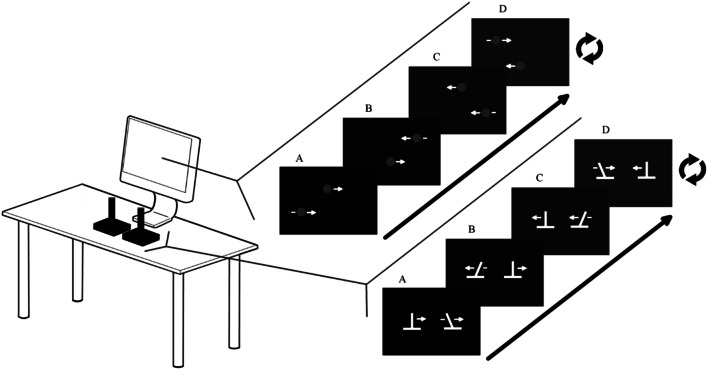
Experimental setup: action sessions. Participants use both joysticks to control the horizontal movements of the dots on the computer display. The visual display on the computer screen (a) corresponds with the position of the joysticks (a). The figure shows an example of moving at 90°. This is achieved by moving linearly from a to d and repeating. During the training sessions, moving at 90° ± some error triggers the hot–cold signal in which the white dots turn green (grey in figure) (see “Coordination feedback”)

For Action sessions, participants used two USB Logitech Extreme 3D Pro joysticks. The central spring and the rubber guard were removed to disable force feedback (see Fig. 1). The vertical position of both dots on the screen was fixed, but the horizontal position of both dots were controlled by the horizontal position of the joysticks, with the left and right joystick corresponding to the top and bottom dots, respectively. The mapping of the joysticks to screen amplitude is set so that required amplitude on the screen does not entail hitting the limits of the joystick range of movement. This forces participants to actively control the joysticks as much as possible, rather than to simply slam into the joystick endpoint to stop.

For Judgment sessions, the participant responded to displays using a USB keyboard. Responding with the “A” and “L” keys for the first and second choice, respectively.

### Procedure

Participants performed between 9 and 13 separate sessions on separate days (see Table 1). The exact number of sessions performed by each individual participant was dependent on when various criteria were met during training (see Criteria). During the Baseline assessment session, participants performed three different tasks (two Action, one Judgment) in the order described (approximately 45 min to complete). In the Post-Training and Retention assessment sessions, participants repeated the procedure from Baseline with one additional perturbation Judgment session described below (approximately 60 min to complete). Participants completed the Baseline, Training and Post-Training sessions within a 3 week time frame, and completed the Retention session 14–24 days after the Post-Training session. Each training session took approximately 20 min to complete.

**Table 1:** Experimental design

Baseline 1 session	5 × 20 s trials each of bimanual 0°, 180°, 90° Criterion for participation: 90° < 0° & 180°; 90° < 0.50 5 × 20 s trials each of bimanual 30°, 60°, 120°, 150°
	2AFC judgment task (90°)
Training	30 × 20 s trials bimanual 90° w/feedback ± 30°
6–10 sessions	30 × 20 s trials bimanual 90° w/feedback ± 25°
	30 × 20 s trials bimanual 90° w/feedback ± 20°
	30 × 20 s trials bimanual 90° w/feedback ± 15°
	30 × 20 s trials bimanual 90° w/feedback ± 10° 30 × 20 s trials bimanual 90° w/feedback ± 10°
Post-training	5 × 20 s trials each of bimanual 0°, 180°, 90° 5 × 20 s trials each of bimanual 30°, 60°, 120°, 150°
1 session	2AFC judgment task (90°) 2AFC judgment task (perturb position)
Retention 1 session	5 × 20 s trials each of bimanual 0°, 180°, 90° 5 × 20 s trials each of bimanual 30°, 60°, 120°, 150° 2AFC judgment task (90°) 2AFC judgment task (perturb position)

All participants worked through these tasks in the order noted. The feedback bandwidth (e.g. ± 30°) indicates over what range from the target phase, the colour feedback is triggered. This is faded over time to drive learning (Wilson et al., 2010a, 2010b). See “Criteria” regarding the performance-based progression employed

### Action task (assessment sessions)

All participants were shown an 8 s, 1 Hz demonstration of the target relative phase (0°) and performed one 20 s practice trial of producing that relative phase at 1 Hz with the joysticks. Participants then performed one block of four 20 s trials in which they controlled the horizontal motion of both dots. The top dot was controlled by the left hand, the bottom dot by the right hand. Participants were instructed to move the joysticks in a smooth, side-to-side, movement to produce the target phase at 1 Hz. This block structure was then repeated for 180° and 90° relative phase, in that order.

These data were used to ensure that none of the participants were already able to perform 90° at a level equivalent to 0° and 180° and could take part in the study (see Criteria). After this, participants performed a second set of coordinated rhythmic movements to measure Baseline performance at 30°, 60°, 120° and 150°, using the same structure as above.

### Judgment task

Following the action tasks, participants performed a series of two-alternative forced choice (2AFC) judgments for 90°. 2AFC is a standardised psychophysical measure for determining perceptual thresholds (see Snapp-Childs et al., 2015; Wilson & Bingham, 2008; Wilson et al., 2010 for applications to coordination perception).

Each trial started with a 4 s demonstration trial of 90°, followed by the presentation of a pair of successive displays. Both displays contained two dots moving harmonically on the screen at some mean relative phase, for 4 s at 1 Hz. The dots were centred on the screen, with an amplitude of 300 pixels (~ 11.5 cm). Of each pair, one showed two dots moving at 90°, and the other was different from 90°; the order was randomly selected on each trial. The task for the participants is to choose which one of the displays shows 90° (pressing ‘A’ for the first and ‘L’ for the second, with no speed requirement).

How different the two displays were was determined using two independent but interleaved transformed 1-up/2-down staircase procedures. One staircase controlled the different displays less than 90°, one for those greater than 90°. Both used a step size “up” of 10° and a stop rule of 8 reversals. Step size “down” was fixed to 54.88% of the step size ‘up’ according to Table 5.1 of Kingdom & Prins (2009); here 5.48°. The initial difference for each staircase was set to 30° and trials only stepped down until the first reversal (first error), after which the staircase procedure was applied. Participants are given knowledge of results (KR) after each trial (“Correct!” or “Incorrect!”). This procedure is essentially identical to that used in Snapp-Childs et al. (2015) with the addition of the KR.

In the Post-Training and Retention sessions, participants repeated the 2AFC task and then completed an additional 2AFC task in which a position perturbation is applied to the display (Wilson & Bingham, 2008). In these displays, the amplitude of the top dot is changed at random on every half cycle, with the constraint that the dot must cross the midline of the screen and cannot exit the screen. The amplitude of the bottom dot is then set to half the top dot’s amplitude, so that it varies randomly but in a way that is coupled to the other dot—this preserves the relative phase. Where and when peak amplitude and peak velocity occur, therefore, change on every half cycle. This preserves mean relative phase (and relative direction information about that relative phase), while making it impossible to use relative position information to perceive relative phase, because there is no stable information about where the dots are within their cycles. We applied this perturbation to replicate Wilson & Bingham (2008) who have already shown that learning 90° entails learning to perceive relative phase via relative position.

### Action task (training)

Following Baseline assessment, participants were trained to bimanually produce 90°. The number of training sessions completed by each participant depended on their performance (see Criteria). The number of training sessions across participants varied between six and ten.

During each training session, participants performed thirty 20 s trials where their goal was to produce 90°. Participants received *coordination feedback* for all trials except for every fifth trial (Wilson et al., 2010). This feedback changed the colour of the dots from white to green when performance was within the given error bandwidth of the target relative phase. In the first training session, the error bandwidth is set at ± 30° and was reduced by ± 5° across sessions when the Criterion for Progression was met (to ± 25°, ± 20°, ± 15°, ± 10°). This colour feedback was not present in the assessment action tasks; or that reason, coordination feedback is removed every fifth trial to help prevent dependence on it (Kovacs, Buchanan, & Shea, 2009; Snapp-Childs et al., 2015).

After every trial with feedback, participants also received KR feedback based on their performance, in which the participant is given a performance percentage (their PTT20 score as a percentage) and a comment (see Table 2). Finally, participants received additional KR at the end of each training session in the form of a level-progression statement. This simply stated whether or not the participant would stay at the current level or progress to the next level. We found that this helped participants stay on task and remain motivated through the extensive training.

**Table 2:** Knowledge of results (performance-generated score)

Performance	Comment
<25%	This is still a little low—keep trying!
25–50%	Definitely improving—keep it up!
50–75%	Doing great—keep it up!
>75%	This is really great—great job!

### Criteria

Prior to training, all participants’ 90° production was substantially worse than 0° and 180° (Mean PTT20: 0.22; 0.77; 0.81, respectively). Participants were then trained in accordance with several predefined criteria (see preregistration). In each training session, when PTT20 was greater than 0.5 in at least 20/30 trials, the participant progressed to the next training stage. This was used to confirm that the participant was ready for progression and to avoid occasional poor performance trials from halting progression. Meeting this criterion resulted in the feedback bandwidth of the next training session to be reduced by ± 5°; otherwise the feedback was kept the same. Training was stopped if PTT20 was greater than 0.6 in at least 20 trials for the last two training sessions (feedback bandwidth at ± 10°), or when participants completed ten training sessions. Participants completed between six and ten training sessions. All participants progressed to and completed at least one session with the feedback bandwidth set to ± 10°.

## Data analysis

### Judgments

For the Judgment tasks, the computer recorded the responses (“correct” or “incorrect”) in relation to the relative phase of the “different” displays that were shown. We separately averaged the difference from 90° of relative phases at which reversals in the staircase procedure occurred for the “different” phases that were greater than 90° and those less than 90°, excluding the first reversal, for each participant. We then averaged those thresholds for each participant.

### Movement

The raw movement data are a 60 Hz time series of the position of the joysticks over time. Each time series was centred on 0, filtered with a low-pass Butterworth filter (cut-off frequency 10 Hz), and differentiated to compute the velocity time series. The continuous-phase time series of each joystick was computed as the arctan(V/X) for each data point and the difference between these time series was the relative phase time series. We then computed the proportion of this time series that fell within 20° of the target relative phase (PTT20).

### Contrast analyses

To analyze transfer of learning, we used Dependent Measures Contrast Analyses (Rosenthal, Rosnow, & Rubin, 2000). This analysis allows us to test for a specific hypothesized pattern of differences across multiple means with a single test (rather than the less powerful and less targeted method of an ANOVA followed by pairwise comparisons). In this experiment, we applied a contrast analysis to performance in the three assessment sessions at each untrained relative phase in which we tested for the specific pattern of change observed at the trained relative phase of 90°.

The test statistic, *t*, is computed as

1 $$t_{\text{contrast}} = \frac{{\bar{L}}}{{\sqrt {\frac{{\widehat{\sigma }_{\text{L}}^{2} }}{n}} }} {\text{with }}\quad L_{i} = \mathop \sum \limits_{j}^{k} (x_{ij} \cdot \lambda_{j} ),$$

where *x* is the data and *λ* are weights. The *λ* weights are the way of quantifying the hypothesised pattern, here set by assessment session performance at 90° (see below). If the data do not differ in the specific way implemented by the Lambda weights (*λ*), then *L*(*i*) is near to zero [i.e. *H*_0_ is *L*(*j*) = 0]. In terms of transfer, a statistically significant *L*(*i*) score for data at a particular untrained relative phase indicates that the specific pattern of improvement observed at 90° is also occurring at that particular untrained phase; the learning has transferred.

## Results

We first examined performance across assessment sessions at 90° to identify whether and how participants had improved with the training. We used the identified pattern of learning to set the *λ* weights for the contrast analyses, and examined the other six relative phases (0°, 30°, 60°, 120°, 150° and 180°) to identify whether the observed pattern of learning at 90° had transferred to any of these conditions. Finally, we repeated this basic analysis plan with the judgment data.

### Learning

**Fig. 2:**
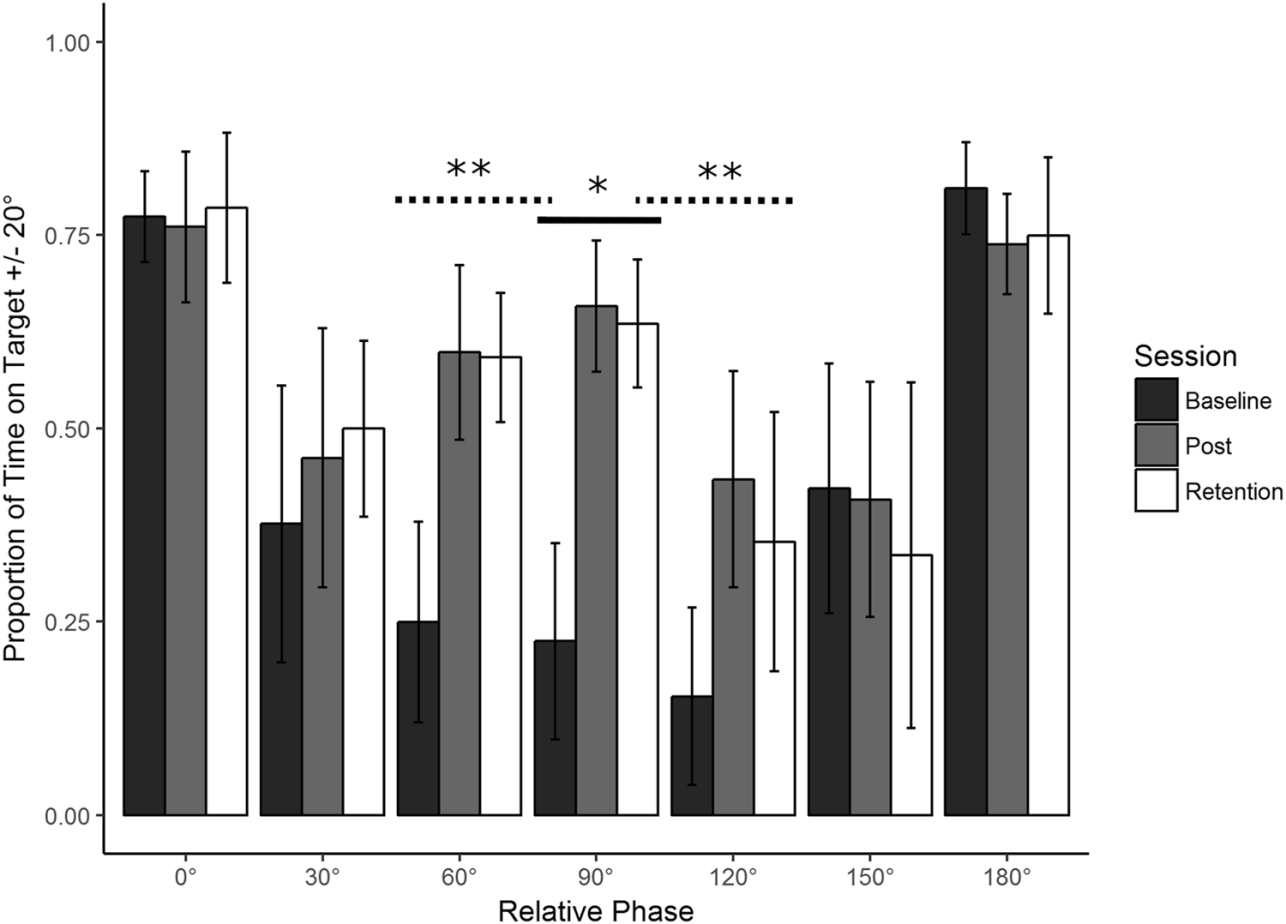
Average performance data (Proportion of Time on Target ± 20°) with standard error bars for all phases in the three assessment sessions (Baseline, Post-Training and Retention). There was a significant main effect of Session for the trained phase of 90° (*). This learning transferred to 60° and 120° (**, see the “Transfer” section for further detail)

Refer to Fig. 2. To examine whether and how training at 90° changed performance at 90°, we analyzed average PTT20 using a one-way repeated-measures ANOVA with Session (Baseline, Post-Training,^3^ Retention) as a within-subjects factor.

Participants significantly improved their coordination stability from Baseline to Post-Training and that learning was retained to Retention. Specifically, there was a significant main effect of Session, *F*(1.12, 10.08) = 87.35, *p* < 0.001. Bonferroni adjusted post hoc analyses revealed a significant difference between Baseline and Post-Training, *t*(9) = − 8.611, *p* < 0.001, MD = 0.433, Baseline and Retention, *t*(9) = − 12.725, *p* < 0.001, MD = 0.411, but not between Post-Training and Retention, *t*(9) = 0.986, *p* > 0.05, MD = 0.022.

The observed learning pattern in performance was ‘worst at Baseline, better and equally so at Post-Training and Retention’. We, therefore, set the *λ* weights for the Action data at − 2 for Baseline, 1 for Post-Training and 1 for Retention. This was done in accordance with the guidelines set by Rosenthal, Rosnow, & Rubin (2000).

### Transfer

No explicit predictions were made regarding where transfer was likely to take place. We, therefore, ran a linear contrast analysis on performance across assessment sessions for all six criterion tasks (0°, 30°, 60°, 120°, 150°, 180°), with a Bonferroni corrected target *α* = 0.0083. A significant contrast analysis, using the above *λ* weights, would show that the same pattern of learning at 90° was present in the criterion task.

**Fig. 3:**
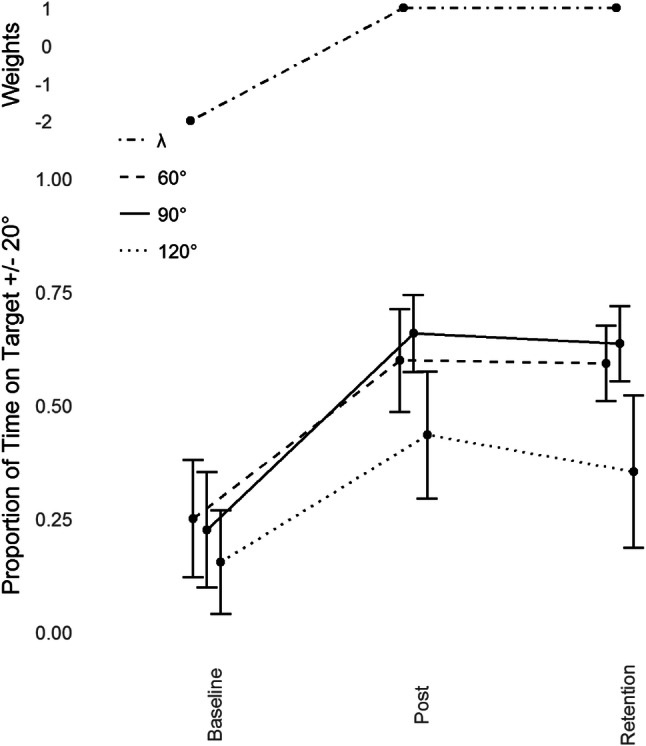
Average performance data (Proportion of Time on Target ± 20°, lower) with standard error bars (lower) for the trained phase of 90° and its transfer partners 60° and 120° in the three assessment sessions (Baseline, Post-Training and Retention) with corresponding Lambda (*λ*) weights (upper)

Refer to Figs. 2 and 3. Dependent Measures Contrast Analyses revealed significant transfer to 60°, *t*(9) = 6.968, *p *< 0.001, *g* = 2.203 and 120°, *t*(9) = 5.116, *p* < 0.001, *g* = 1.617. No transfer was detected at any other phase (0°, 30°, 150°, or 180°, all *p* > 0.05).

We calculated the proportion of transfer across all conditions by taking the difference between Post-Training and Baseline performance for the criterion task, and dividing that by the difference between the Post-Training and Baseline performance for each of the transfer tasks. This assesses both the direction and magnitude of any change in the transfer task, proportional to the changes of the criterion task. Performance at 0°, 150° and 180° worsened as a function of practice at 90°. This decrease was minimal at 0° (− 3%) and 150 (− 3%) but larger at 180° (− 16%). There was some increase of performance at 30° (19%), but only substantial increase at 60° (81%) and 120° (65%), all of which aligns with the results of the contrast analyses. Transfer was also asymmetric; it was considerably greater at 60° than 120°, although both effects were large in magnitude

### Judgment thresholds

**Fig. 4:**
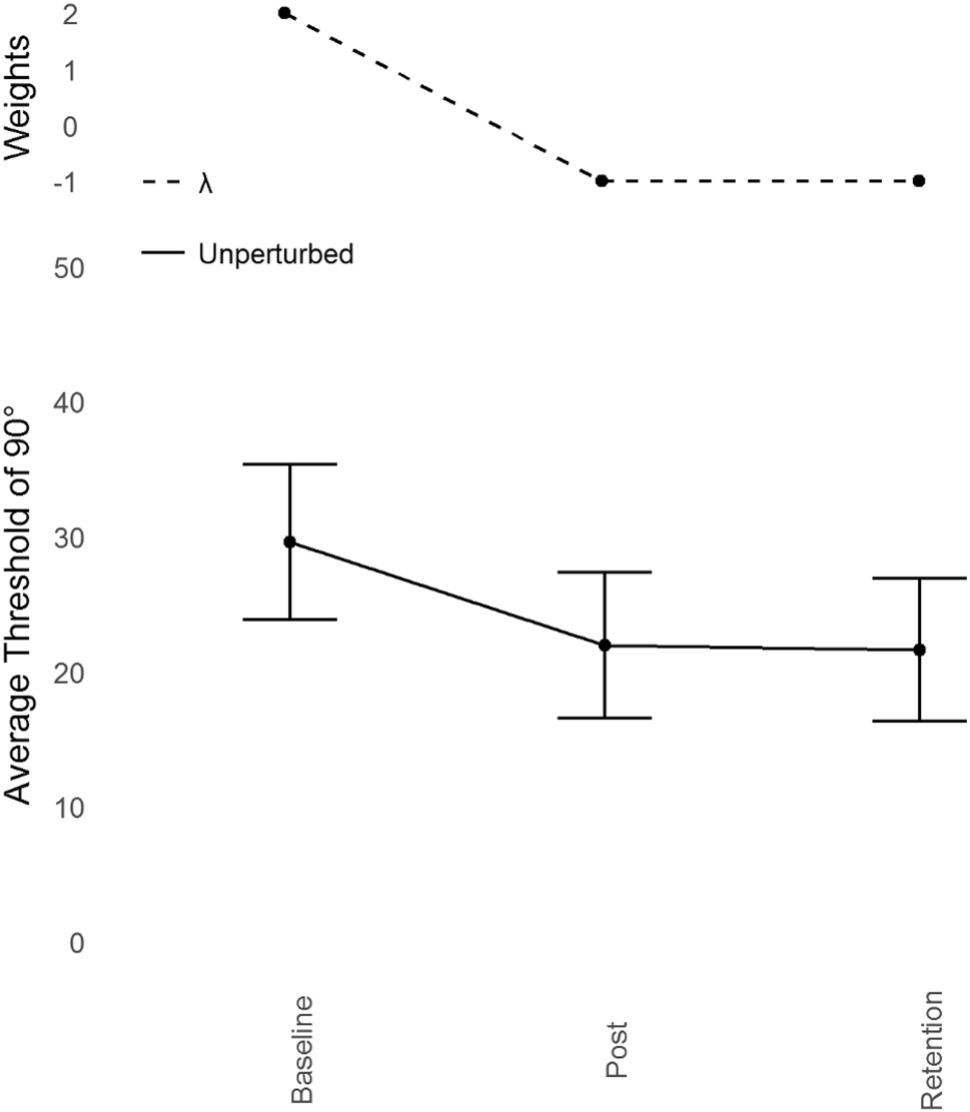
Average unperturbed perceptual judgment thresholds for 90° (lower) with standard error bars at Baseline, Post-Training and Retention with corresponding Lambda (*λ*) weights (upper)

Refer to Fig. 4. Learning a coordinated rhythmic movement primarily entails learning to perceive the target novel relative phase, which in turn allows stable coordinated actions (Wilson et al., 2010). We, therefore, predicted that the judgment data would mirror the learning data in the action task (as in Snapp-Childs et al., 2015).

Prior to any training, thresholds for identifying which display showed 90° were high (*M* = 29.61°, SD = 5.74°). After training, this threshold improved (*M* = 21.93°, SD = 5.42°) and remained lower perfectly after the Retention period (*M* = 21.61°, SD = 5.31°).

#### Contrast analyses

To test the prediction that the judgment data mirrors the learning data in the Action task (90°), we used the *λ* weights identified in the learning pattern of 90° to predict the same pattern in the judgment data. The lower the threshold, the greater the ability to discriminate between the target relative phase (90°) and other relative phases. Thus, the sign of the *λ* weights are reversed to comply with the nature of the measure (2 for Baseline, − 1 for Post-Training, − 1 for Retention). A Dependent Measures Contrast Analysis with the within-subjects factor of Session (3 levels; Baseline, Post-Training and Retention) and the dependent variable of unperturbed judgment thresholds of 90°, revealed a significant effect with a large effect size, which suggests that the Action-driven *λ* weights are a good fit for the judgment data, *t*(9) = 4.801, *p *< 0.001, *g* = 1.518.

#### Unperturbed and perturbed judgment threshold comparison

**Fig. 5:**
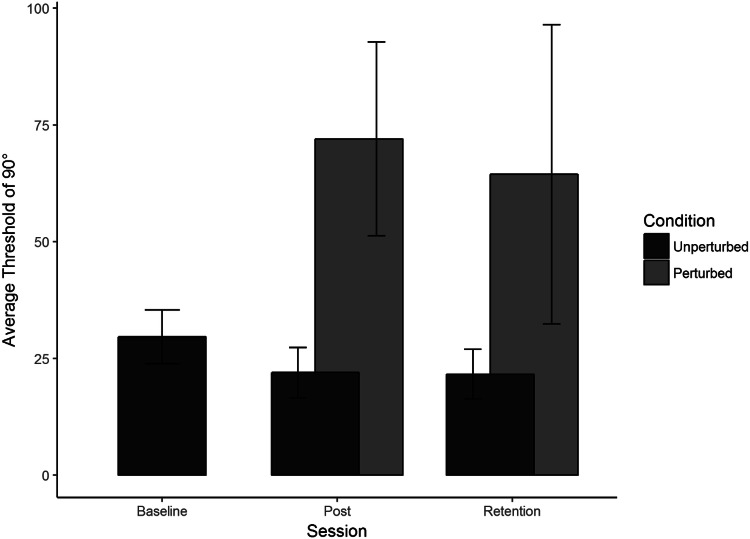
Average perceptual judgment thresholds for 90° with standard error bars at Baseline, Post-Training and Retention. There was a significant main effect of condition, with the perturbation reducing performance. The contrast analysis demonstrated that the learning data were a good fit for the unperturbed judgment data

Refer to Fig. 5 (for averages and individual data, respectively). Thresholds for identifying 90° were lower than Baseline in the unperturbed condition in both Post-Training (*M* = 21.93°, SD = 5.42°) and Retention (*M* = 21.61°, SD = 5.31°) but were extremely high and variable in the perturbed condition for both Post-Training (*M* = 71.99°, SD = 20.74°) and Retention (*M* = 64.41°, SD = 32.1°). Participants improved perceiving and moving at 90° by switching to using relative position, and when this was no longer informative about relative phase, they could no longer do the Judgment task.

To compare the unperturbed judgment thresholds at Post-Training and Retention with the perturbed judgment thresholds, we performed an ANOVA on average judgment thresholds with condition (Unperturbed and Perturbed) and Session (Post-Training and Retention) as factors. There was a significant main effect of condition, *F*(1, 36) = 56.86, *p* < 0.001, with no other significant main or interaction effects.

### Exploratory data check: bias analysis

The dependent variable PTT20 has been used in both this and previous experiments (see, Snapp-Childs et al., 2015) to ask questions regarding transfer of learning using an error bandwidth of ± 20°. During the early phases of training, the feedback was triggered over a wide range (± 30) and this was then reduced according to performance. One potential issue is that what we have reported as transfer (say to 60°) could simply be a bias towards one particular instance of the feedback. For example, when presented with the task of moving at 90° an individual might spend time moving at 75°, which is within our PTT20 threshold for ‘on target’ for both 90° and 60°.

We repeated the transfer analysis with a reduced bandwidth of ± 10°. If bias towards a particular instance of the feedback is what is driving the transfer effect then the results should be characteristically different. That is, the performance landscape should look different. However, if PTT20 is successfully capturing transfer, the results will replicate (likely with a reduced effect, as performing within ± 10° requires a higher degree of accuracy).

The learning pattern found with PTT20 at 90° was identical with a reduced bandwidth of PTT10. Participants improved their coordination stability from Baseline to Post-Training and that learning was retained. An ANOVA confirmed this with a main effect of Session (F (1.22, 10.9) = 81.02, *p* < 0.001). The difference between Baseline and the other assessment sessions was driving this effect (both *p* < 0.001), and there was no significant difference between Post-Training and Retention (*p* > 0.05).

As the learning pattern was the same at PTT10, the weights for the contrast analysis were set the same (− 2, 1, 1). The pattern of transfer found with the reduced bandwidth mirrors what was found at PTT20, with reduced effect sizes (yet still large in magnitude). The learning at 90° transferred to 60° (*t*(9) = 6.88, *p* < 0.001, *g* = 2.17) and 120° (*t*(9) = 4.96, *p* < 0.001, *g* = 1.57) and nowhere else (*p* > 0.05).

This analysis tells us two things. First, the pattern of transfer we observed was not caused by any systematic bias in how people performed during training. Second, it confirms that the 20° bandwidth for the PTT20 measure is appropriate; the bandwidth does not dictate the pattern of results (see also Wilson et al., 2010, who checked bandwidths of 10°, 15° and 30° and found the same result).

## Discussion

This study had two parts; evaluating the mechanism of learning to move at 90° (what changed in the participants that supported learning?) and whether that mechanism supported stable movement at any other relative phases (did the learning transfer?).

Based on previous work, we hypothesized that learning to bimanually perform 90° would be enabled by improved visual discrimination of 90°, in turn enabled by the switch to the variable relative position. Unperturbed judgment data confirmed improved visual discrimination of 90°, and the perturbed judgment data confirmed that people were perceiving 90° using relative position (replicating previous work; Wilson & Bingham, 2008; Wilson et al., 2010). Thus, learning to perform 90° relative phase entails a switch in information use from relative direction to relative position.

Does this information switch support stable production at other relative phases (transfer of learning)? Performance at two untrained relative phases (60° and 120°) saw a considerable increase after training at 90° and this increase was still present 2–4 weeks later. Thus, for the first time in the coordination literature, we see substantial transfer of learning from one relative phase to another that is not a symmetry partner. Cross-effector transfer of the same relative phase has been observed (Kelso & Zanone, 2002; Snapp-Childs et al., 2015), and these types of transfer effects can be large (up to 50%). However, the percentage transfer that we see here from 90° to 60° (81%) and 120° (65%) is larger still. The perceptual learning of relative position that occurred at 90° has enabled a greater increase in performance at other relative phases than merely changing the limbs performing the task.

The nature of the feedback we use in training is, therefore, critical. The colour change coordination feedback preserves all the informational consequences of two coordinated oscillators, one of which (relative position) can support stable behavior at other relative phases. Visual metronomes and Lissajous displays do not preserve this information; instead, they convert the task into either a discrete or continuous tracking task and, in the case of Lissajous displays, integrating the relative motion information for coordination into a single motion source. Visual metronomes do not support any transfer across relative phases, and Lissajous displays make all relative phases equally easy to produce with minimal practice (Kennedy et al., 2016), making the issue of transfer redundant. While these other feedback methods still have their uses, our results demonstrate that we must always consider tasks such as coordination as perception–action tasks, and take issues of feedback information very seriously.

### Asymmetry of transfer

The Bingham model is of an untrained system that uses relative direction to perceive relative phase. The model is still informative here, however. Learning transferred asymmetrically; performance at 60° improved more than performance at 120°. In the model, relative speed acts as a non-phase specific noise term that affects a person’s ability to detect the underlying information for relative phase (Snapp-Childs et al., 2011). This allows the model to implement the fact that 180° is less stable than 0°, and the fact that coordination stability decreases with increases in frequency. Relative speed increases linearly from 0° to 180°; this tidily accounts for the asymmetry we observed (and the bias check we performed supports this). At this point, we, therefore, assume that relative speed affects the detection of relative position in the same way it affects the detection of relative direction; this remains to be explicitly tested, however. That future work should apply the methods from Snapp-Childs et al. (2011) to trained performance at various relative phases.

### Location of transfer

Learning 90° transferred to two neighbouring relative phases. A dynamical systems’ account (c.f. Kelso, 1995; Zanone & Kelso, 1994) might simply suggest that learning 90° creates a new attractor centred on 90° but relatively spread out. However, simple proximity is not an explanation in and of itself; it is the thing to be explained. In addition, this does not provide an obvious explanation for the asymmetry of transfer we observed.

We have shown that the relative position variable learners attune to when training at 90° supports stable action close by, but not farther away. However, we also know that relative position can support stable behavior at 0° and 180° without improving untrained performance at 90° (Wilson & Bingham, 2008). This pattern suggests that the specific calibration of relative position used at various phases might differ. For example, 90° is specified by relative position as the relative phase when the points of peak amplitude and peak velocity occur at the same time (this is what the position perturbation makes impossible to use, because this positional alignment happens at different locations of every half cycle). 0° and 180° are the relative phases when the points of peak amplitude occur at the same time. It is possible that these different calibrations may be sufficiently different to prevent transfer. More work is clearly needed to probe the exact nature of the relative position variable and its possible calibrations, by training other relative phases with coordination feedback and examining the pattern of transfer, and by investigating the consequences of the individual variation in variable use identified in Wilson & Bingham (2008). These studies are ongoing in our lab.

### Direct learning

The goal of this work is to more completely characterize relative position so that it can be implemented in a version of the Bingham model that directly engages with learning and trained performance. Ecological psychologists have been recently grappling in more detail with the idea that multiple information variables can be informative to varying degrees about the underlying task dynamics. Learners often seem to begin using lower order, non-specifying variables (e.g. speed, distance) that are informative within a limited scope, and then switch to using higher order specifying variables (e.g. time-to-contact) after training with feedback (see Withagen & Chemero, 2009, for an overview).

This idea has been formalized in the ecological theory of direct learning (Jacobs & Michaels, 2007). In this framework, the possible information variables in a task define an information space, and each possible combination of variables in this space correlates to the task dynamical property to some degree (up to and including the correlation of 1.0 that is specification). As participants attempt to use some variable to perceive and act on the dynamic, they receive information-for-learning that specifies the degree to which the variable is supporting behavior, and the direction in which the learner should move in the information space to improve performance. This guides the education-of-attention towards more useful variables, the use of which can then be calibrated.

Most of the existing research applying this framework depends on dynamic touch tasks, where people wield objects to haptically perceive properties such as relative mass (e.g. Jacobs, Silva, & Calvo, 2009). One problem is that the information spaces here are being defined with respect to dynamical properties (static moment, and the first and third principle moments of inertia) rather than the kinematic information variables specifying those properties. Better work relies on judgments of relative mass after collision events, in which the relevant information variables are known (e.g. Jacobs, Michaels & Runeson, 2000). Our work here lays out an information space for the task of coordinated rhythmic movement. This task can be studied via both judgments and perceptually controlled actions, and so we now have a more comprehensive paradigm for investigating both the education-of-attention and calibration direct learning processes, as well as transfer of that learning.

## Summary

The current study used task-appropriate feedback to investigate learning and transfer of learning in coordinated rhythmic movement. For the first time, we have shown that transfer of learning to other relative phases is possible, and that this transfer is supported by switching which information variable people are using to perceive relative phase. This transfer also seems to be affected by the relative speed of the oscillators acting as a noise term on the detection of the information variable.

This study is part of our ongoing efforts to expand the Bingham model of coordination dynamics, so that it can also model learning and trained performance in the task. This model and the empirical research it is driving emphasize the perception–action nature of skilled action, with the particular goal of characterizing the perceptual information and how that is used in the context of the task. Learning, and transfer of learning, can only be fully understood from this perspective, and while there is much still to do, we now have a comprehensive task analysis and a set of experimental tools (action, perceptual judgment and perturbation methods) to develop a more complete account of performance and learning in coordinated rhythmic movement.

## Footnotes

1 Specifically, de Rugy et al. tested the bimanual model prediction with a unimanual coordination task, and analysed performance using a measure of coordination variability. Snapp-Childs et al adapted the model to capture unimanual (visuomotor) coordination and analysed performance with a more valid measure, proportion-time-on-target. The more appropriate test confirmed the hypothesis.

2 We split the sample according to the training-set criterion into learners (*N* = 4) and non-learners (*N* = 6) and analysed average PTT20 using a repeated-measures ANOVA with Group as a between subjects factor (Learners and Non-Learners) and Session (Baseline, Post-Training and Retention) as a within-subjects factor. There was no main effect of Group, *F*(1, 8) = 0.972, *p* > 0.05, indicating no difference between learners and non-learners.

3 During one trial in part 2 of the post-training data collection session (trial 14: 120°), participant nine accidently perturbed the monitor wire temporarily disabling the screen. This trial was removed from all subsequent analysis.
